# Perceived stress reduction through an infertility coaching program: a randomized controlled clinical trial

**DOI:** 10.1038/s41598-023-41845-4

**Published:** 2023-09-04

**Authors:** Roghoyeh Soleimani, Fatemeh Ansari, Zeinab Hamzehgardeshi, Forouzan Elyasi, Mahmood Moosazadeh, Fereshteh Yazdani, Maryam Shahidi, Narjes Shiraghaei, Mahtab Karimi, Tayebeh Hemati, Mansooreh Pejmanmanesh

**Affiliations:** 1 Student Research Committee, Nasibeh Faculty of Nursing and Midwifery, Mazandaran University of Medical Sciences, Sari, Iran; 2 Student Research Committee, Mazandaran University of Medical Sciences, Sari, Iran; 3 Sexual and Reproductive Health Research Center, Mazandaran University of Medical Sciences, Sari, Iran; 4 Psychiatry and Behavioral Sciences Research Center, Sexual and Reproductive Health Research Center, Addiction Institute, School of Medicine, Mazandaran University of Medical Sciences, Sari, Iran; 5 Epidemiology, Gastrointestinal Cancer Research Center, Non-communicable Diseases Institute, Mazandaran University of Medical Sciences, Sari, Iran; 6 Shahid Beheshti University of Medical Sciences, Tehran, Iran; 7 IVF Ward, Hazrat-e Maryam Fertility Center (HMFC), Sari, Iran; 8 Women’s Clinic, Nyköping Hospital, Nyköping, Sweden; 9 IVF Ward, The MOM Specialized Centre for Reproductive Health and Infertility, Tehran, Iran; 10 Fertility & Infertility Center, Milad Hospital, Isfahan, Iran

**Keywords:** Psychology, Health care

## Abstract

Infertility has been recognized as a distressing experience among couples worldwide, cutting across various cultures. This present study was conducted to assess the impact of a supportive stress management program led by an infertility coach for women undergoing fertility treatment. This randomized controlled clinical trial study was performed on 60 infertile women undergoing assisted reproductive techniques at Maryam Infertility Center located in Sari in 2018. After random allocation in two groups, 30 individuals were in the intervention group and 30 in the control group. The intervention program was implemented according to the infertility coach's counseling protocol in six stages. The control group received only routine ward counseling. In order to measure stress, the Newton Infertility Stress Questionnaire was used firstly before intervention and then after oocyte puncture, embryo transfer, and pregnancy testing. Data analysis was performed using SPSS statistical software version 18 and Shapiro–Wilk, Chi-square, Mann–Whitney, independent t-test, Friedman test, Wilcoxon test, GEE test, and Cohen's effect size. Our analysis approach has also been based on the analysis of (ITT). The significance level was 0.05. The mean ± SD scores of infertility perceived stress before the intervention in the control was 146.16 ± 16.90 and the intervention group was 156.53 ± 9.31, after intervention at the time of oocyte puncture in the controls was 165.36 ± 8.98 and the intervention group was 155.83 ± 10.70, at the day of embryo transfer in the control group was 156.35 ± 14.45 and in the intervention group was 123.58 ± 22.9 and in the pregnancy test day in the control group was 185.76 ± 26.56 and in the intervention group was 127.61 ± 21.57 (P < 0.001). According to Friedman test, the mean of stress in three situations after the intervention showed a significant difference in reduction of the mean of stress (P < 0.001). In the control group, the stress score of the samples had an increasing trend, which was significant during the measurement steps based on Friedman test results (P < 0.001). In the intervention group, paired t-test results showed no significant comparing mean score of Newton's infertility stress before and after oocyte puncture day (P = 0.711), comparing the mean of stress before and after pregnancy test day (P = 0.003) and also comparing of mean stress before and after pregnancy on the day of embryo transfer according to Wilcoxon test (P < 0.001). And comparing mean stress before and after pregnancy test day, paired t-test (P = 0.001) showed significant statistical differences. According to the results of the GEE test, changes in stress scores over time were significant between the two groups (P < 0.001), as well as the effect of stress on oocyte puncture day (0.41), embryo transfer day (1.69), pregnancy test day (P < 0.001) (2.46) had a significant effect on the day of embryo transfer and pregnancy test day. Based on the results of this study, the infertility coach program demonstrated the ability to decrease the perceived stress related to infertility. Additionally, it showed potential in enhancing treatment outcomes, such as oocyte count and positive pregnancy results, among infertile women undergoing assisted reproductive techniques.

Trial registration: Iranian Registry for Clinical Trial (the link to trial: https://www.irct.ir/trial/33357). Registered 11-11-2018.

## Introduction

Infertility is defined as the absence of pregnancy following a year of unprotected sexual intercourse^1^. The global infertility rate ranges between 12 and 15%^2^. In Iran, based on the clinical, epidemiological, and demographic definitions and criteria provided by the World Health Organization (WHO), the overall prevalence of primary infertility is reported as 20.2%, 12.8%, and 9.12%, respectively. Additionally, the rate of secondary infertility in Iran is estimated to be 4.9%. It should be noted that the prevalence of infertility in Iran exceeds that at a global level^3^.

The experience of infertility, which some people call the infertility crisis, is associated with physical, social, and economic stress that affects all aspects of a person's life^2^. Studies have shown that the inability to cope with stress activates the immune response in the individual and causes failure of the implantation process. In other words, long-term stress and reduced ability to adapt are significantly associated with a high proportion of activated T-cells in the peripheral blood, which reduces the implantation rate in women undergoing in-vitro fertilization (IVF)^4^.

The technological nature of assisted reproductive techniques (ARTs) creates a stressful atmosphere. Patients enrolled in ART programs often experience sadness and frustration caused by infertility and appear depressed, angry, tired, dependent, anxious, and emotionally deficient. But when couples are introduced to ARTs, hope that did not exist until a few years ago is revived.

They find themselves in a new emotional turmoil of conflicting feelings of hope and despair that seems to be part of the nature of technology itself. Given the stressful nature of ARTs, it is clear that patients need psychological support as part of the medical treatment process. This support can be provided by a coach accompanying them throughout the treatment process. The ART treatment team can have a variety of responsibilities, including patient coaching, which is more likely to make patients satisfied with their treatment and more likely to accept the outcome^5^.

It was also recommended in the fields of reproductive health and infertility to improve the treatment results of ARTs, it is better to use complementary methods such as coaching to reduce the stress and anxiety resulting from these methods, and subsequently, increase positive outcomes^6^.

The coach boosts self-management skills in making sensitive decisions related to infertility treatment, patient awareness, information recall, and participation in decision making. Patients' participation in the treatment process leads to greater satisfaction and trust and lower anxiety and stress^7^. There is a growing consensus among all reproductive organizations that a patient-centered approach is needed to respect the needs and values of the patient and respond to all his clinical concerns and decisions. In a patient-centered approach^8^, the presence of an infertility coach in all stages of ART treatment and responding to counseling needs during the stages of infertility treatment can reduce the patient's stress, and thus, improve the results of ARTs.

Currently, there is a global consensus that infertility treatment centers should provide essential counseling programs for managing the mental health challenges among infertile people^9^. Therefore, due to the lack of a comprehensive study on the impact of an infertility coaching program on the perceived stress of infertile women and the outcome of treatment, the researchers decided to perform this study to investigate the effect of an infertility coaching program on perceived stress in infertile women and treatment outcome of ARTs.

## Methods

### Study design

The current study was designed as a randomized controlled parallel clinical trial aimed at examining the impact of an infertility coaching program on the perceived stress levels of infertile women undergoing ARTs. Experimental protocol/s was approved with the code 1671.1397 IR.MAZUMS.REC by the Ethics Committee of Mazandaran University of Medical Sciences.

The allocation ratio was 1:1. The participants of the present study were infertile women treated with ARTs who visited Hasrat-e-Maryam Infertility Treatment Center in Sari in 2018. It should be mentioned that the women visiting this center are from all cities across Mazandaran province and the neighboring provinces.

The random allocation sequence was generated using a random-numbers table as the method for this process. The samples were randomly (lottery) divided into the two groups of intervention (n = 30) and control (n = 30). Using sequentially numbered, opaque, sealed envelopes was the mechanism used to conceal the sequence until interventions were assigned. That informed consent was obtained from all the patients/legal guardian during the study. All patients who provided consent for participation and met the inclusion criteria were randomized. The process of randomization was carried out by the designated staff member responsible for recruitment. The assessors responsible for evaluating the outcomes remained blinded to the treatment assignments.

IRCT registration number: IRCT20180811040762N1. Due to the start of repairs in one of the centers during sampling, multi-center sampling was updated to a single center, and Changes were made to the previous revision. (https://www.irct.ir/trial/33357?revision=113962).

### Inclusion criteria

Inclusion criteria consisted of women with primary infertility, women treated with ARTs (maximum 3 times), age less than 45 years, Iranian nationality, literacy, no intention to use a donated embryo or ovum or rent a womb, no adopted child, no counseling by a psychologist or psychiatrist at the time of the study or at least one month before the start of the study, no major psychiatric disorder at the time of the study (initial diagnosis by a psychiatrist) and no use of psychiatric medications, non-use of cigarette, hookah, alcohol, or anti-müllerian hormone (AMH more than 1.5 ng/mL), and no systemic diseases such as diabetes based on treatment history, patient files, and report of the participants.

### Exclusion criteria

Exclusion criteria comprised of ovaries’ failure to respond to ovulation-inducing drugs, cessation of ovulation induction therapy, cancellation of IVF Spouses’ level of education program Spouse’ employment, IVF for any reason under the supervision of a physician, death of a loved one or accident during treatment, and occurrence of pregnancy diagnosed by blood test during the study.

### Sample size

The sample size was calculated based on the study of Yazdani et al.^10^ and using G-power software. In the mentioned study, the mean and standard deviation of oocyte count in the intervention group were 12.5 and 6.3, respectively, and in the control group were 8.64 and 3.91, respectively. Taking into account these results and 95% confidence level and test power of 80% and using the formula for comparison of two means in G-power software, the sample size was calculated at 48 people (24 people in the intervention group and 24 people in the control group). Considering 20% sample attrition, 6 people were added to each group. Thus, the total sample size was calculated to be 60 people.

### Procedure

**Figure 1:**
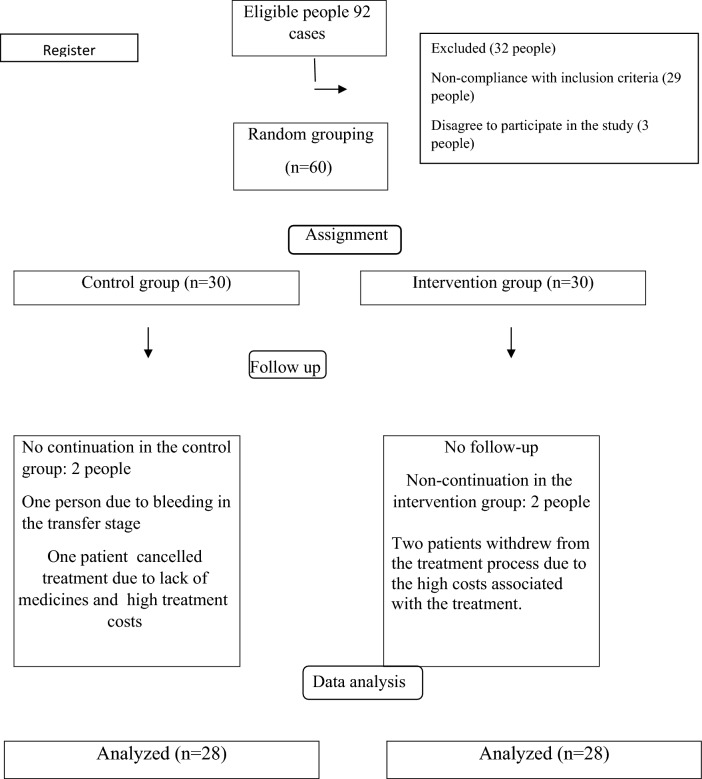
Flow diagram of the study method.

Prior to the study intervention, the participants of the intervention group (after completing the consent form) were given explanations about the purpose of the program, time, method of counseling, and study procedure. Then, the consent form and the demographic, general health, and Newton stress reduction questionnaires were completed by the patients before the intervention. Information on demographic and general health questionnaires is shown in Tables 1 and 2. All questionnaires were completed by the researcher during individual interviews in a separate room. The intervention was performed from July 2017 to March 2018. The flow diagram of the study method was shown in Fig. 1.

**Table 1:** Frequency of demographic characteristics.

Variables	Group	Control	Intervention	P-values
		No. (%)	No. (%)	
Age	20–29	9 (30)	9 (30)	P = 0.864
	30–39	20 (66.6)	19 (63.3)	
	40–45	1 (3.3)	2 (6.6)	
Woman’s level of education	Diploma or less	15 (50)	15 (50)	P = 0.602
	BSc	14 (46.7)	11 (36.7)	
	MSc or higher	1 (3.3)	4 (13.3)	
Spouse’ level of education	Diploma or less	17 (56.7)	17 (56.7)	P = 1.000
	BSc	13 (43.2)	13 (43.2)	
	MSc or higher	0 (0.0)	0 (0.0)	
Woman’s employment	Housewife	17 (68)	19 (79.2)	P = 0.472
	Employed	8 (32)	5 (20.8)	
Spouses’ employment	Employee	12 (48)	15 (62.5)	P = 0.506
	Self-employed	11 (44)	6 (25)	
	Laborer	2 (4)	3 (12.5)	
Cause of infertility	Female	11 (44)	10 (41.7)	P = 0.263
	Male	2 (8)	5 (20.8)	
	Mutual	4 (16)	6 (25)	
	Unknown	8 (32)	3 (12.5)	

The test used to compare the variables in this table is chi-square or Fisher's exact test. Significant level was set to less than 0.05.

**Table 2:** Sessions description of study intervention.

Session	Before visit	Session content	Session duration	After visit	Session duration
First session	The first visit of women	Information on the anatomy of the reproductive system and hormones related to fertility, a brief explanation of the stages of treatment with assisted reproduction methods, answers to questions and concerns of the research unit about how to treat, consequences and possible complications	min:30	Answers to possible questions after the visit	min:15
Second session	Days 19 to 20 of the cycle	Information on how stress and negative emotions affect hormonal and physiological changes in the reproductive system, training to control negative emotions, training on strategies to strengthen sperm and ovum	min:30	Answers to possible questions after the visit	min:15
Third session	Day 2 of the next cycle (approximately 10 days after the second session)	Training to improve communication skills	min:30	Answers to possible questions after the visit	min:15
Fourth session	Day 6–8 of the cycle (about one week after the third session)	Teaching stress reduction techniques and effective coping strategies	min:30	Answers to possible questions after the visit	min:15
Fifth session	Ovum retrieval and around day 14 of the cycle	Brief description of ovum retrieval	min:30	Answers to possible questions after the visit	min:15
Sixth session	Transfer day	A brief explanation of the transfer operation	min:30	Answers to possible questions after the visit	min:15

The Newton Stress Reduction Questionnaire was completed at the pre-puncture, pre-embryo transfer, and pregnancy test stages by the patient. Necessary consultations and interventions were performed during the treatment process.

The content of the sessions was presented in six sessions before and after the patient visit and each session lasted for 45 min. The content of the meetings is shown in Table 3. In order to check the validity of the content of the sessions, 10 experts (4 reproductive health specialists, 2 gynecologists, 3 psychiatrists, and 1 psychologist) provided their opinions, which were reviewed and applied.

**Table 3:** Comparison of the mean perceived stress of infertility at three time points after the intervention and over time in the control and intervention groups.

Mean stress	Intervention group		Control group		Significant level of comparison between the two groups (independent t-test/Mann–Whitney	Effect size	Significant level of comparison of stress score changes over time between the two groups (GEE test)	
	Mean	SD	Mean	SD		Intervention	Intervention	Control
Total stress before	156.53	9.31	146.16	16.92	0.05	–	0.000	0.000
Stress the whole day of ovum retrieval	155.83	10.70	165.36	8.98	0.000	0.41		
Stress throughout the day of embryo transfer	123.58	22.92	165.35	14.45	0.000	1.69		
Stress the whole day of the pregnancy test	127.61	21.57	185.76	26.56	0.000	2.46		
Significant level of comparison of stress score of total day of ovum retrieval with stress score before intervention (paired t-test)	0.711		0.000					
Significant level of comparison of total stress day of embryo transfer with pre-intervention stress score (Wilcoxon test)	0.000		0.021					
Significant level of comparison of total day stress score of pregnancy test with pre-intervention (Paired t test)	0.000		0.000					
Significant level of comparison of stress score over time in each group (Friedman test)	0.000		0.000					

*GEE* generalized estimating equations.

This support program was conducted during individual counseling sessions by a midwifery counseling graduate after training by the research team. In the control group, the questionnaires were completed by the patient. They received only the routine care provided by the center.

### Data collection tools

1. Demographic Questionnaire consists of the two sections of personal and family information and infertility information, which includes personal and family information including information on age, education, spouse education, employment status, and spouse employment status, place of residence, duration of the marriage, history of a previous marriage of the wife, and previous marriage history of the spouse and menstrual status. In the section on infertility information and its treatment, the duration of infertility, treatment duration, infertility cause, and hope for treatment success were asked.
2. General Health Questionnaire: This is a self-report screening questionnaire that is used clinically to track those who are prone to mental disorders^11,12^. It examines a person's mental health during the last month. A lower score indicates better mental health^12^. In Iran, Taghavi et al. have established the validity and reliability of this questionnaire^13^. In this study, this questionnaire was used to assess the general health status of infertile patients.
3. Newton Stress Reduction Questionnaire: The primary outcome was assessed using this questionnaire.

Newton Infertility Stress Questionnaire is a reliable self-report questionnaire with appropriate validity and reliability^14^. Therefore, this tool can be used to diagnose infertility stress. The Newton Infertility Stress Questionnaire is a 46-item tool designed by Newton in 1995 and consists of five subtests including social concerns (10 questions), sexual concerns (8 questions), communication concerns (10 questions), rejection of life without children (8 questions), and the need to become a parent (10 questions). This questionnaire is rated based on a 6-point Likert scale. The overall score of the questionnaire is between 46 and 276, with higher scores indicating more stress^14^. This questionnaire was administered in Iran in 2005 by Alizadeh et al. to establish its reliability among a sample of 30 infertile people (15 males and 15 females), and the obtained Cronbach's alpha coefficients for the subscales of social concerns, sexual concerns, communication concerns, rejection of childless lifestyle, and need for parenthood were respectively 0.78, 0.77, 0.78, 0.75, and 0.84, and the Cronbach's alpha coefficient for overall stress was 0.91^15^. Permission to use this questionnaire was obtained from Dr. Farahani (corresponding author).

### Statistical analysis

Data analysis was performed using SPSS statistical software version 18.

In the current study, the independent variable is the infertility coaching intervention, while the dependent variable is the level of perceived stress of infertility. To describe the variables under investigation, various statistical measures were employed, including means, standard deviations, medians, and frequency percentages.Then, to examine the normal distribution of the data Shapiro–Wilk, Chi-square, and Fisher's exact tests were used to compare the categorical variables between the intervention and control groups. To compare the mean stress scores at the first, second, and fourth time points between the intervention and control groups, the independent t-test was used. Additionally, to compare the mean stress score at the third time point, the Mann–Whitney U test was employed. Generalized estimation equation (GEE) test was used to compare the mean changes in stress score over time (four measurement steps) between the two groups.

Also, to compare the mean stress score on ovum retrieval, embryo transfer, and pregnancy test days with pre-intervention times in the intervention and control groups, paired t-test and Wilcoxon test were used. It should be noted that the trend of stress score changes over time (four measurement steps) in each group was determined by the Friedman test. Our analysis approach was based on analysis for intention to treat. Cohen’s effect size was also used to examine the difference between the mean scores of stress. P-A value of less than 0.05 was considered statistically significant**.**

### Ethics approval and consent to participate

This study with the code 1671.1397 IR.MAZUMS.REC was registered at the Ethics Committee of Mazandaran University of Medical Sciences. This study was also registered with the code IRCT20180811040762N1 in the IRCT system.

## Results

### Demographic profile findings

Table 1 presents the demographic characteristics of the participants. Importantly, there were no significant differences observed between the intervention and control groups concerning various demographic factors. These factors included variables such as age, educational attainment, educational level of spouses, employment status, employment status of spouses, underlying cause of infertility, duration of marriage, length of time of infertility awareness, and duration of treatment.

### Findings of perceived infertility stress

The results of this study showed that the mean and standard deviation of perceived infertility stress score before the intervention was 146.16 ± 16.92 in the control group and 156.53 ± 9.31 in the intervention group (P = 0.05). The mean perceived stress after the intervention on the day of ovum retrieval was 165.36 ± 8.98 in the control group and 155.83 ± 10.70 in the intervention group (P = 0.000). Mean perceived stress after the intervention on the day of embryo transfer in the control group was 165.35 ± 14.45, while it was 123.58 ± 22.92 in the intervention group (P = 0.00), (P = 0.00), and on the day of pregnancy test this score was 185.76 ± 26.56 in the control group and 127.61 ± 21.57 in the intervention group (P = 0.00). The mean perceived infertility stress was significantly different between the two groups after the intervention (Table 3).

Based on the results of Friedman test, stress score in the intervention group at the three stages after the intervention showed a significant reduction (P = 0.000). In the control group, the stress score of the samples showed a significant rising trend based on the results of Friedman test (P < 0.000) (Table 3). The results of paired t-test by comparing the mean score of Newton infertility stress before and after the day of ovum retrieval (P < 0.711), comparing the mean of stress before and after the day of pregnancy test (P < 0.003), and also comparing the mean of stress before and after the day of embryo transfer according to Wilcoxon test (P < 0.000) showed a statistically significant difference (Table 3).

Based on GEE test results, considering that the difference in baseline stress score between the two groups was statistically significant, after adjusting the effect of the baseline stress score, the stress score changes over time between the two groups were significant (P < 0.001). The effect size of stress on the days of ovum retrieval, embryo transfer, and pregnancy test was 0.41, 1.69, and 2.46, respectively, which was significant on the day of embryo transfer and day of pregnancy test (Table 3).

**Figure 2:**
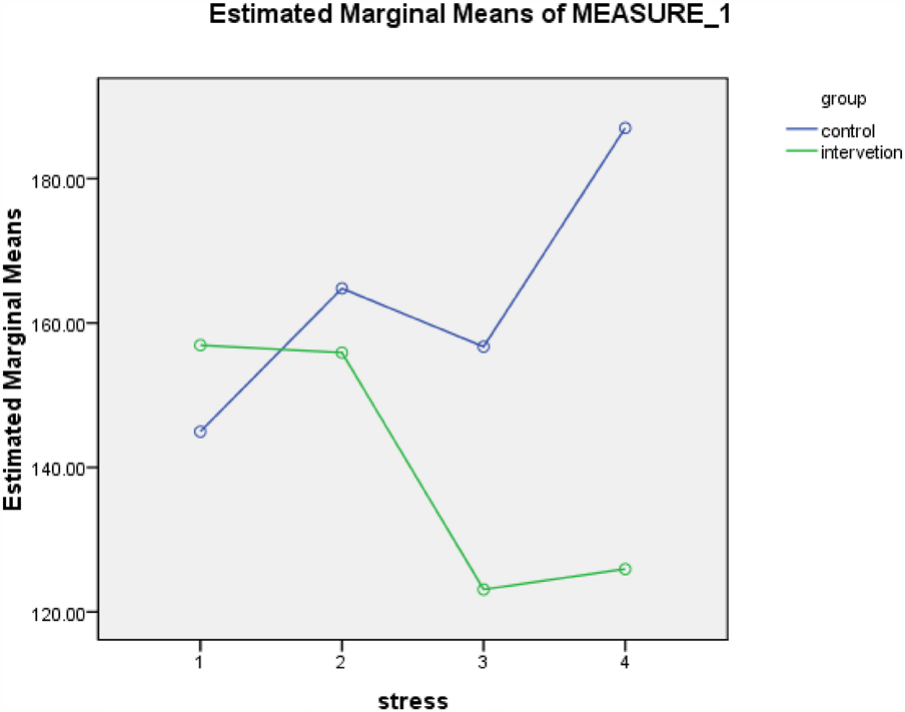
Comparison of mean and standard deviation of Newton's infertility stress score in infertile women, separated into two intervention and control groups, in three situations before and after the intervention on the days of egg collection, embryo transfer and pregnancy test.

Figure 2 showd the comparison of mean and standard deviation of Newton's infertility stress score in infertile women, separated into two intervention and control groups, in three situations before and after the intervention on the days of egg collection, embryo transfer and pregnancy test.

## Discussion

The aim of this study was to determine the effect of an infertility coaching program on perceived stress in infertile women undergoing ARTs. The findings of the present study showed that the presence of an infertility coach can mitigate stress in infertile women. The interventions performed in the intervention group had an effect on the stress perceived on the days of ovum retrieval, embryo transfer, and pregnancy test, such that the stress in these three stages was reduced compared to the stages before the intervention. In the control group, the amount of stress in the stages of ovum retrieval, embryo transfer, and pregnancy test day was significantly increased compared to before the outset of the study.

The results of this study are in line with the study of Latifnejad et al. 2011 (participatory counseling)^11^, the study of Cassill et al. (role of supportive group therapy for short stress management)^16^, and study by Faramarzi et al. (cognitive-behavioral therapy)^17^, Mosalanejad et al. (cognitive-behavioral therapy)^18^, Yazdani et al. (group counseling)^10^, Kheirkhah et al. (the effect of counseling on infertility adjustment)^19^, Boivin (the effect of psychological interventions)^20^, Chan et al. (psychosocial counseling)^21^, and Domar et al. (internet-based mindfulness counseling)^22^. It can be concluded that various types of counseling (group counseling, educational and psychological counseling) can reduce the perceived stress resulting from infertility, which can indicate that infertile people are under physical, mental, and social, stress and are in dire need of support, counseling, and training in fertility treatment.

In the study of Latifnejad et al.^11^, the effect of participatory infertility counseling on the perceived stress of infertile women undergoing IVF treatment was statistically significant. The results of Faramarzi et al. showed that the use of cognitive-behavioral techniques in controlling depression and anxiety was more effective than pharmacotherapy (fluoxetine). Also, follow-ups performed 1 and 3 months after the intervention showed the permanent effects of these methods^17^.

A study was performed by Yazdani et al. in 2017 to determine the effect of a counseling program provided by a midwife on perceived stress in infertile women and the outcome of assisted reproductive therapy in a clinical trial study. In this study, the intervention group received six two-hour group counseling sessions each week, which were performed by a consulting midwife. The results showed that group counseling by a midwife reduced the perceived stress of infertility and improved treatment outcomes. The results of this study are consistent with the present findings such that midwifery counseling reduced perceived infertility stress and improves treatment outcomes^10^.

Kheirkhah et al. in a clinical trial aimed at determining the effect of group counseling on infertility adjustment in infertile women concluded that group counseling is effective in increasing the adjustment of infertile women undergoing IVF and ICS treatment^19^. Psychological interventions based on counseling cover all psychological dimensions and make a variety of changes in a person's beliefs, feelings, and behaviors, and in most target groups, including patients with cancer, heart attack, and angiography, it reduces patients' anxiety and stress and affects the rates of learning, quality of life, and patient adaptation^19^.

A review study by Boivin, it was aimed to determine the effect of psychological interventions on the mental health and fertility of infertile women and to determine the type of more effective interventions. It was found that psychological interventions were effective in reducing the negative effects of infertility and increasing pregnancy. It has been shown that group counseling interventions with an emphasis on educational skills have been more effective than counseling interventions and expression of feelings and support groups^20^.

Chan et al. conducted psychosocial counseling with an approach to Chinese teachings called the Oriental Program to affect the body and soul of women undergoing IVF treatment and reduce their anxiety. The results showed a significant reduction in anxiety levels in the intervention group^21^. Also, in the study of Dumar et al. Internet-based mindfulness counseling showed that Internet-based intervention can reduce anxiety and depression symptoms in infertile women and improve their fertility^22^.

Gurhan’s study showed that counseling by a nurse did not cause a significant difference in the rate of depression between the case and control groups. The counseling sessions were short and consisted of two 20-min sessions by a nurse and one 60-min general session by a nursing faculty member. Due to the fact that IVF treatment is invasive and the grief of these people is more, effective emotional coping skills are necessary in counseling these patients^23^.

The results of the study of Klerk et al. with the aim of counseling in the first period of IVF treatment were not effective. In this study, counseling interventions were presented to patients in three sessions^24^. Hammerli et al. in a meta-analysis that examined the impact of psychological interventions on fertility improvement showed that psychological interventions did not affect mental health, depression, and anxiety. However, these interventions had an effect on infertile women who did not use ARTs, which could be due to the fact that they were less depressed and anxious than those who used ARTs. Also, the treatment duration of those not using ARTs was relatively shorter than those using ARTs (3.5 years vs. 4.5 years). Psychological interventions were more effective in the sexual behaviors of those not using ARTs. In addition, the number of counseling sessions in women without ARTs was greater than the other group (average 3.6 sessions, 9.8 sessions)^25^.

The results of Sexton study showed that Internet-based intervention to deal with infertility stress significantly reduced the general stress symptoms of infertile women, but there was no statistically significant difference between the two groups in terms of perceived infertility stress. Given the nature of the intervention in Sexton's study, it seems that there were many intervening variables in this study that were not well controlled and were beyond the control of researchers and may have affected the reported results. Also, the research units were from the general population of infertile people and no significant results were found in terms of variables such as type of infertility, duration of infertility, current treatment method, number of cases of ARTs, etc. that affect the effectiveness of psychological counseling in women undergoing IVF^26^.

Psychological disorders such as stress cause a vicious cycle that leads to aggravation of problems, and by using counseling programs, infertile people can strengthen their family foundation by taking advantage of desirable marital satisfaction and living without stress and concern. Obviously, the resources and teamwork of gynecologists, midwifery counselors, and psychologist in infertility clinics play an important role in reducing the problems of these patients. In explaining the findings, it should be mentioned that identifying negative future thoughts and replacing them with positive and real thoughts, as well as cooperation of midwives, psychologists and gynecologists in counseling infertile women, is emphasized. Due to the fact that infertile people are affected by psychological and social stress, treatment results are also reduced due to this stress^26^.

Studies related to the effect of coaching programs on diseases such as type 2 diabetes showed that coaching can improve treatment outcomes and adherence to treatment while being cost-effective^27^. It was also recommended in the field of reproductive health. In order to improve the treatment results of assisted reproductive methods, complementary methods such as coaching should be used to reduce the stress and anxiety resulting from these methods, and consequently increase positive results^6^. The findings of this study using an infertility coaching program and providing protocol-based counseling showed that perceived infertility stress was diminished in infertile women undergoing ARTs.

In various studies, it has been tried to reduce stress in infertile women using various ways. It should be mentioned that to improve treatment outcomes, a combination of supportive and educational counseling with the infertility treatment team, including a trained midwife specializing in midwifery counseling due to the midwifery clinical basis and a good understanding of the field of counseling, can be powerful support during the treatment of infertile women undergoing ARTs.

## Conclusions

The present study investigated the effect of an infertility coach as a supporter and counselor during infertility treatment on infertile women’s perceived stress. Due to the negative effects of stress and the existence of many new problems in treatment with ARTs such as lack of awareness of the treatment process, lack of support, lack of constant access to the treatment team, invasiveness of ARTs, and heavy treatment costs, these people face more challenges that reduce the possibility of successful treatment. It seems that the presence of a knowledgeable coach who can accompany the patient throughout the treatment process can reduce the level of stress. Therefore, the use of an accompanying coach along with the ARTs treatment team in the relevant centers is recommended due to the positive results of this study is significantly reducing stress in infertile women undergoing ARTs.

### Study strengths

One of the strengths of this study is that according to the research team, this study is the first interventional study of infertility coaching by a counseling midwife with the aim of reducing the perceived stress of infertile women undergoing ARTs. Also, in the present study, the structure of the intervention was established by presenting a protocol developed by the expert team. According to sample size, a procedure for sampling, and the participation rate, these study’s results are broadly applicable to many infertile women and different types of infertility centers and situations.

### Study limitations

Due to the difficult economic situation and the dramatic increase in treatment costs and sometimes disruption in the provision of drugs and equipment used in ART treatment, some of these patients faced challenges prolonging their treatment process. Thus, it was not possible to evaluate the results of their treatment at this time.

## Data Availability

All the methods were performed in accordance with relevant guidelines and regulations 31 codes of ethics in Iranian biomedical research and the Declaration of Helsinki. The datasets used and/or analyzed during the current study are available from the corresponding author on request.
